# Single-shot CT after wrist trauma: impact on detection accuracy and treatment of fractures

**DOI:** 10.1007/s00256-018-3097-z

**Published:** 2018-11-08

**Authors:** Monique Brink, Arjan Steenbakkers, Micha Holla, Jacky de Rooy, Simon Cornelisse, Michael J. Edwards, Mathias Prokop

**Affiliations:** 1 Department of Radiology and Nuclear Medicine, Radboud University Medical Centre Nijmegen, huispost 780, PO Box 5601, 6500 HB Nijmegen, The Netherlands; 2 Department of Surgery, Radboud University Medical Centre Nijmegen, Nijmegen, The Netherlands; 3 Department of Orthopedics, University Medical Center Groningen, Groningen, The Netherlands

**Keywords:** Wounds and injuries, Multidetector computed tomography, Radiography, Wrist Injuries, Emergency radiology, Casts, Surgical

## Abstract

**Objective:**

To evaluate accuracy of fracture detection and therapeutic impact of a single-shot CT protocol as a primary imaging tool in all patients with clinical suspicion of wrist injury, and evaluate the resulting impact on therapy.

**Materials and methods:**

We performed a single-institution study on all patients with suspicion of fractures of the wrist and carpus. All patients underwent conventional radiography, thereafter single-shot wrist CT, and then 1-year follow-up. Physicians and radiologists prospectively scored likelihood of fracture presence on a five-point scale before and after CT. Three surgeons proposed a treatment regimen (functional, cast, reduction, or operative) based on clinical and radiological data, first with knowledge of conventional radiography, and then with knowledge of CT. The reference standard for fracture presence was based on all data. We performed receiver operating characteristic (ROC) analyses and calculated proportion of wrists with treatment changes due to CT imaging.

**Results:**

Ninety-eight patients participated (63% female, mean age 53, range, 18–87 years old) with 100 wrist CTs. Conventional radiography detected true-positive fractures in 45, and CT in 61 wrists. The areas under the curve for fracture detection were 0.85 (95% CI 0.77–0.93) for conventional radiography and 0.97 (95% CI 0.93–1.00) for CT. Treatment changed in 24 (24%, 95% CI 16–33%) - 31 (31%, 95% CI 23–41%) wrists, mostly involving a decrease in the rate of cast immobilization.

**Conclusions:**

Single-shot CT in patients with clinical suspicion of wrist injury increases accuracy of fracture detection. This has a significant impact therapy in this population, mainly on cast immobilization.

**Trial Registration:**

We registered the study at www.clinicaltrials.gov, NL43482.091.13.

## Introduction

Wrist fractures are very common injuries, and their prevalence increases with age in the Western population [1]. These injuries not only cause pain and disability but also represent a large economic burden, with both high health-care costs and productivity loss [2]. Optimal treatment can prevent malunion or nonunion, avascular necrosis, and post-traumatic osteoarthritis [3–5].

Conventional radiography (CR) is the standard modality in cases of suspicion of radiocarpal injury. However, CR underestimates the presence of intra-articular distal radius fractures [6] while fractures of the scaphoid and other carpal bones are often unnoticed on CR [5]. For this reason, patients with clinical suspicion of scaphoid injury and a negative CR either undergo cast immobilization [7] or receive more advanced cross-sectional imaging such as computed tomography (CT) or magnetic resonance imaging (MRI) [8, 9] after CR. This frequently changes treatment in selected patient populations, especially in case of intra-articular distal radius fractures and pre-operative planning [10–12] and might be cost-effective in case of scaphoid injury suspicion and a negative CR [13].

CT scanning techniques have currently been optimized by decreasing artifacts, radiation exposure, and imaging times [14]. In practice, this makes CT a more accessible modality, providing high contrast three-dimensional information on bone surfaces. We implemented a low-radiation dose wrist CT protocol in clinical practice that takes approximately 30 s patient time in the scanning room: Single-shot CT. This protocol includes a fast setup, no table movement, and an effective dose of less than 0.02 mSv.

The purpose of this study was to evaluate accuracy of fracture detection and therapeutic impact of this fast set-up CT protocol as an imaging tool in all patients with clinical suspicion of wrist injury, and evaluate the resulting impact on therapy.

## Materials and methods

### Patient selection

We performed a single-institution, prospective cohort study with patients who attended our center for evaluation of the distal radius and ulna or the carpal bones after trauma. Between June 5, 2013 and April 29, 2014, all consecutive patients who fulfilled the inclusion criteria were eligible for study participation.

Patients of 18 years of age or older were eligible if they were scheduled for conventional radiography because of clinical suspicion of wrist trauma not older than 3 days. Patients were excluded in case of (1) no physical evaluation by a physician before imaging, (2) open fractures, (3) patients could not be positioned in upright position because of immobilization on a spine board or transfer to the intensive care unit, (4) no informed consent or no prospective data collection could be obtained.

### Study procedures

Patients were screened by requesting physicians, radiologic technologists, and (resident) radiologists for eligibility before they underwent imaging at the Department of Radiology and Nuclear Medicine. After informed consent, anteroposterior and true lateral conventional radiography images of the wrist on a flat-panel CR system (DelftDI Trauma CR, Canon, Benelux) were obtained. Additional targeted carpal views were only acquired if requested. Radiology residents supervised by musculoskeletal or emergency radiologists reviewed and immediately reported all CR views on a picture archiving and communication system (PACS) and communicated their findings to the requesting physicians.

In case of fractures with displacement, physicians performed reduction, casting, and new CR after reduction. The physician thereafter prospectively completed a CT request form with findings and confidence level at CR (likelihood of fracture presence and likelihood of presence of intra-articular components of the distal part of the radius on a five-point scale: not present, probably not present, equivocal, probably present, present) in consensus with the reporting radiologist. This form had to be completed before CT was executed.

Thereafter, the patient underwent single-shot CT on an Aquillon Vision One 320-slice CT scanner (Canon Medical Systems, Otawara, Japan) in an upright position, sitting on a chair next to the CT table and leaning on the CT table with the affected arm in pronation.

A volume scan with a maximum of 320 slices was performed without table movement, no scanogram, 0.5-s rotation time, either 12-, 14-, or 16-cm volume length depending on the length of the wrist and carpus, pitch 0, 80-kV tube voltage, a fixed tube current time of 50 mAs, and iterative reconstruction. Standard multiplanar reconstructions were automatically executed in axial direction with a bone kernel and a soft tissue kernel, a section width of 0.5 mm and an increment of 0.3 mm, and in coronal and sagittal direction with a bone kernel, a width of 2 mm and an increment of 1 mm. Standard 3D surface shaded reconstructions and reconstruction along the long axis of the scaphoid were reconstructed at the discretion of the radiologist.

The radiologist interpreted the CT images and reported the final diagnosis to the physician in a written report and by telephone. Based on all findings, physicians decided on a treatment plan, and followed patients according to clinical practice. All radiological reports were generated according to a structured report that was implemented at the beginning of the study. These reports included information on image quality, presence of fractures, likelihood of fracture presence and radiocarpal intra-articularity on five point scales, presence and extent (in mm) of displacement, and presence of soft tissue injury.

### Clinical data collection and data handling

Two researchers collected data on mechanism of trauma, clinical data (patient age, sex, type of trauma, relevant medical history, clinical findings, findings at CR and CT, treatment plans), follow-up on presence of fractures, operations and complications from the electronic patient files and research forms. For additional follow-up, all patients were asked to complete a validated Web-based questionnaire on the disability of arm, shoulder, and hand (patient-rated wrist and hand score (PRWHE)) score [15]) at four time points: during the initial emergency visit, and 6 weeks, 6 months, and 1 year after trauma. The first questionnaire was considered as a baseline score on complaints before the accident and was completed during the emergency ward visit.

### Standard of reference

One independent trauma surgeon (34 years of experience in traumatology) and one dedicated emergency radiologist (3 years of experience in emergency radiology) assigned the diagnosis of fractures and fracture pattern based on all available information after follow-up: clinical information, radiological requests, images and reports, surgery, follow up, and questionnaires. In case of discrepancy, consensus was reached in a panel with three radiologists and one surgeon.

### Treatment changes

In order to evaluate the impact of CT on treatment choices, a retrospective observer study was performed with three experienced trauma surgeons (25, 15, and 19 years of experience) in two sessions.

During the first session, observers proposed a treatment plan after CR. They received anonymous, standardized information on mechanism of trauma, patient characteristics, and CR findings. They could simultaneously review anonymous CR images on a PACS system, but were blinded to other information such as CT findings, actual treatment, and follow-up.

During the second session, at least 2 months later, observers proposed a treatment plan after reviewing the same information as in the first session, with addition of the CT findings.

Observers could choose between four different treatment regimens: Functional treatment (no immobilization, pressure dressing) on the emergency ward, conservative treatment with cast without closed reduction, treatment with cast with closed reduction, or operative treatment.

### Statistical analysis

In case of missing data on presence of symptoms or fractures, we considered these features to be absent. Missing data due to follow-up loss were not included in the outcome analysis. We performed receiver operating curve (ROC) analyses based on the prospectively collected likelihoods (1-5) of presence of fractures and presence of intra-articularity of distal radius fractures on CR and CT compared to the standard of reference. We calculated the true-positive, false-positive, false-negative, and true-negative rates for different fracture locations. Difference in intra-articular distance of bony fracture fragments was calculated using the paired sample* t* test.

The main study parameter was the proportion of patients with treatment changes after CT. For each observer, the proportion of patients with treatment changes, upgrades (change from more invasive treatment towards less invasive or aggressive treatment), and downgrades (change from invasive treatment towards less invasive after CT) were calculated, including 95% confidence intervals. Observer agreement was calculated with Fleiss-weighted kappa analysis [16]. The intended sample size was 100 wrists, as we considered a 95% confidence interval of 20% around the estimated proportion of treatment changes acceptable. Statistical calculations were executed with SPSS (SPSS, IBM, version 22.0.0.1, New York, USA) and Microsoft Office Excel 2007 (Microsoft, Redmond, WA, USA).

## Results

**Fig. 1:**
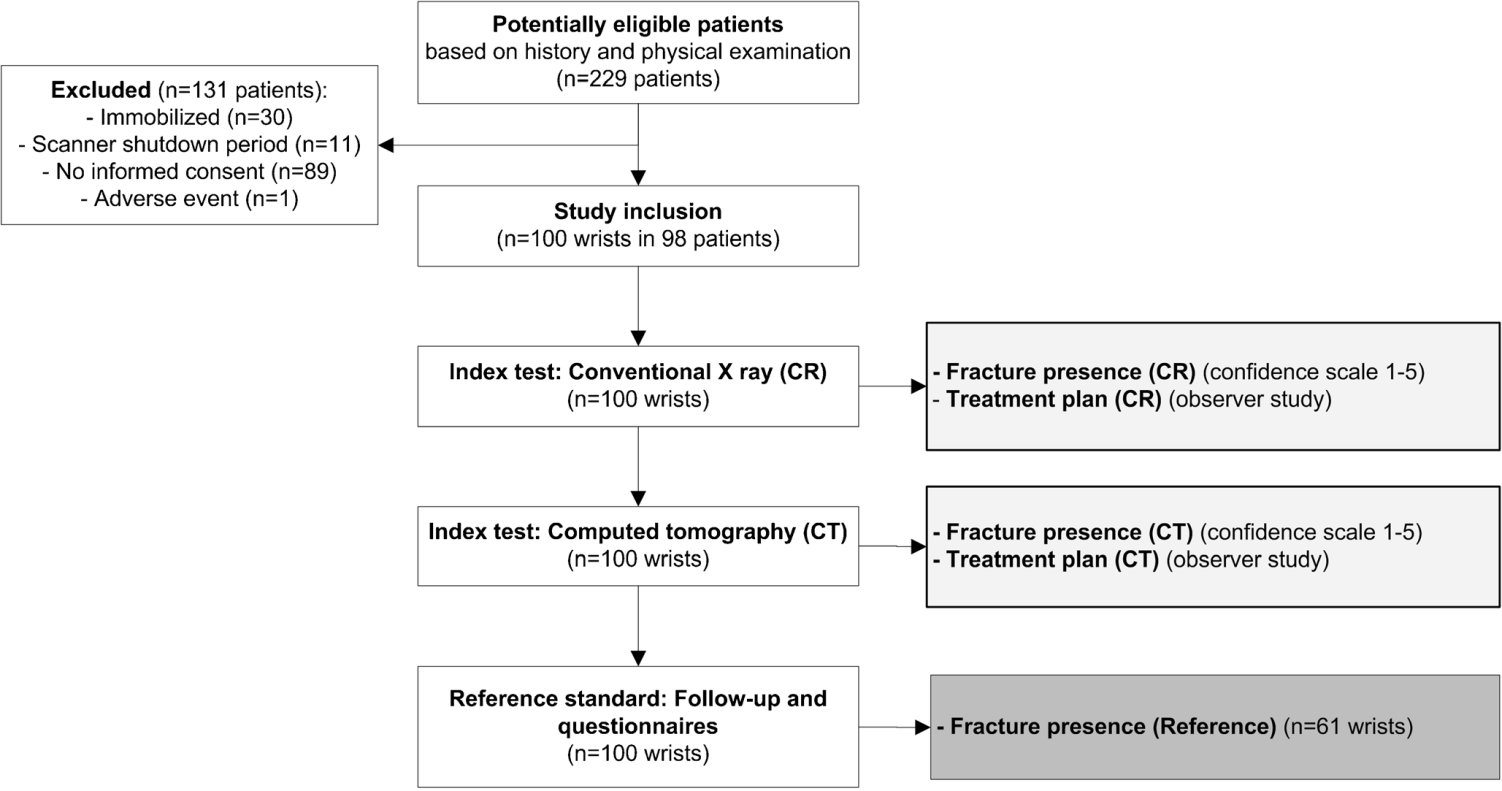
Flowchart of patient inclusion and data selection of the main outcome parameters

Between June 5, 2013, and April 29, 2014, 229 patients were eligible, of whom 98 patients with 100 wrists were included (Fig. 1). One patient was included for evaluation of both wrists and one patient was included twice, each time evaluated for a single, but different wrist. Median age was 53 years (range, 18-87), 37 patients were male and 61 patients were female.

### Index test fracture detection

Of all 100 CR investigations, additional targeted oblique CR views were taken in 40 wrists, with scaphoid views performed in 34 patients. CR detected fractures in 51 wrists (see Table 1).

**Table 1:** Accuracy of fracture detection of the wrist including fracture locations, of conventional radiography (CR), and computed tomography (CT) as compared to the standard of reference

	CR						CT					
	CR TP	CR FP	CR FN	CR TN	Sens	Spec	CT TP	CT FP	CT FN	CT TN	Sens	Spec
Fractures	45	6	15	34	75%	85%	61	1	0	38	100%	97%
Radius	39	2	2	57	95%	97%	41	0	0	59	100%	100%
Ulna	13	1	1	85	93%	99%	14	0	0	86	100%	100%
Carpus	6	2	20	72	23%	97%	26	1	0	73	100%	99%
Scaphoid	2	3	6	89	25%	97%	8	0	0	92	100%	100%
Triquetral	2	0	9	89	18%	100%	11	0	0	89	100%	100%
Lunate	0	0	3	97	0%	100%	3	0	0	97	100%	100%
Trapezium	0	0	6	94	0%	100%	6	0	0	94	100%	100%
Trapezoid	0	0	1	99	0%	100%	1	0	0	99	100%	100%
Hamate	1	0	0	99	100%	100%	1	0	0	99	100%	100%
Capitate	0	0	0	100	100%	100%	0	1	0	99	100%	0%
Metacarpal	2	0	1	97	67%	100%	3	0	0	97	100%	100%

*TP* true positive, *FP* false positive, *FN* false negative, *TN* true negative, *Sens* sensitivity, *Spec* specificity

Of all 100 single-shot CT scans, 16 were performed after closed reduction in cast. CT detected fractures in 62 patients (Table 1).

### Standard of reference

**Fig. 2:**
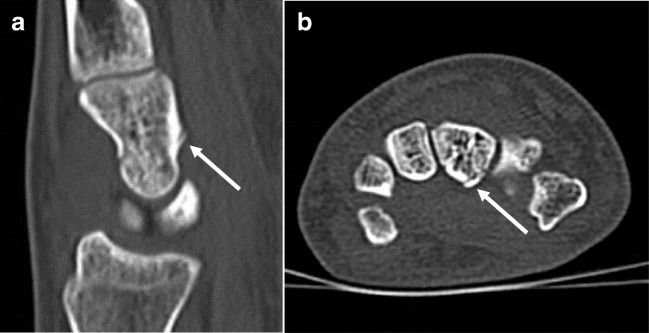
False-positive CT of the wrist of a 32-year-old woman who fell on her left arm in dorsiflexion. At physical examination, she had excoriations and pressure pain at the level of the dorsal radius. There was no fracture at CR. Although at CT there was suspicion of a fracture of the capitatum (**a** and** b**,* arrow*), the patient was treated with a pressure dressing because she refused cast treatment. At follow-up, pain decreased within 1 day. In retrospect, the finding at CT was caused by a vascular channel, without any visible swelling or induration in the surrounding soft tissue

Forty-five patients (45%) responded to the Web-based questionnaires. Median PRWHE was 28 after 6 weeks (43 patients) and 10 after 1 year (37 patients). In 61 wrists, one or more fractures were present according to the standard of reference: A total of 41 fractures in the distal radius, 14 in the distal ulna, and 25 in one or more carpal bones. In one patient with suspicion of scaphoid bone injury, CT was false positive. This case is illustrated in Fig. 2.

**Fig. 3:**
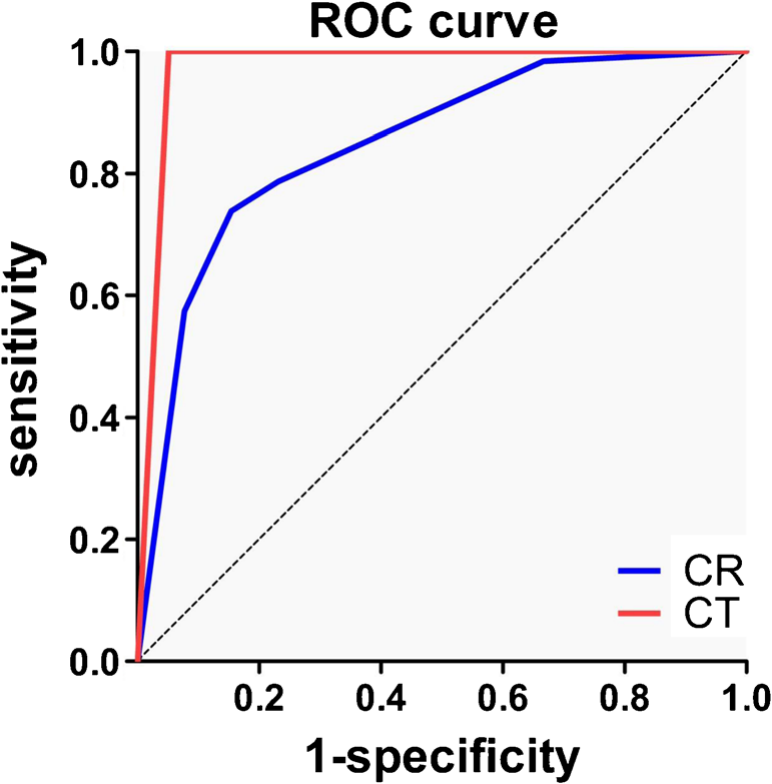
Receiver operating characteristic (ROC) curve for fracture detection as compared to the standard of reference. Area under the curve was 0.85 (95% CI 0.77-0.93) for conventional radiography (CR,* blue line*) and 0.97 (95% 0.93–1.00) for single-shot computed tomography (CT,* red line*)

CT detected additional fractures or different relevant fracture patterns compared to CR in 26 wrists: 26 carpal fractures in 21 wrists, two distal radius fractures, one distal ulna fracture, and seven intra-articular fractures. Seven of these patients had additional targeted oblique views before CT. Table 1 displays accuracy numbers for all types of fractures for CR and CT as compared to the standard of reference. Area under the curve was 0.85 (95% CI 0.77–0.93) for CR and 0.97 (95% 0.93–1.00) for CT (Fig. 3).

Of all wrists with radial fractures (*n* = 41), 32 had a radiocarpal intra-articular component according to the standard of reference. Area under the curve for this intra-articularity detection was 0.87 (95% CI 0.77–0.96) for CR and 0.96 (95% CI 0.91–1.00) for CT. Of all wrists with an intra-articular gap, this was measured 1.2 mm at CR and 2.5 mm at CT (*p* < 0.05).

### Impact on patient treatment

In clinical practice, 23 wrists underwent functional treatment, 57 cast immobilization only, 15 closed reduction and cast, and five patients underwent surgery. Two were operated on because of (carpo-) metacarpal fractures, two underwent external fixation, and one underwent screw fixation because of a delayed union of a scaphoid fracture despite immediate cast immobilization. In two patients, an operation was proposed, but these patients did not consent to this treatment and underwent cast immobilization instead. One patient with a previous history of pseudoarthrosis of the scaphoid underwent an elective screw fixation after several weeks, although this patient did not have an acute fracture at the time of the study according to the reference standard.

In the observer study, agreement on treatment was moderate after CR (Fleiss kappa 0.61 (95% CI 0.51–0.70) and good after CT (0.75 (95% 0.66–0.84)). The number of wrists with treatment changes after CT was 24, a proportion of 24% (95% CI 16–33%) according to observer 2 and 3 and 31 (31%, 95% CI 23–41%) according to observer 1.

**Fig. 4:**
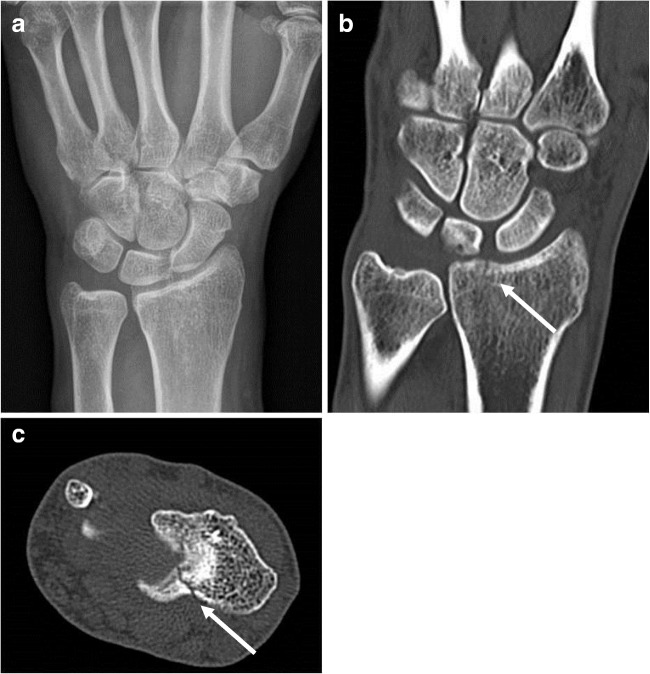
Missed fracture on CR with treatment upgrade after CT. A 45-year-old male with a painful left wrist after fall on an outstretched arm and with swelling at the level of the lunate, but no hematoma or pressure pain. CR was negative for fractures (**a**). Based on these images, both clinicians and the observers proposed conservative treatment with a pressure dressing. CT demonstrated an intra-articular, distal radius fracture (*arrows*,** b** and** c**). All observers upgraded to cast treatment. The patient was finally treated with cast for 3 weeks

Treatment changes after CT are displayed in Table 2 (upgrades) and Table 3 (downgrades). Of all 26 wrists with additional findings, at least one observer upgraded treatment in 11 wrists (11% of the total population, 95% CI 6–18%; 6–8 per observer). These upgrades mainly included more aggressive treatment from pressure dressing to cast because of additional proximal carpal fractures or additional radial fractures (Fig. 4, Table 2). The proposed upgrades were executed in clinical practice in six wrists. The other five patients did not prefer cast, clinicians did not perform reduction, or finally did not consider surgery after 1-week follow-up. None of these patients had complications after follow-up. The other proposed upgrades without additional findings were actually executed in clinical practice in seven patients: The remaining three patients were not operated on because of severe co-morbidity with short life-expectancy in one, and patient refusal in two patients. No downgrades occurred in patients with additional findings on CT.

**Table 2:** Treatment upgrades according to one or more observers

Treatment before CT	Additional diagnoses	Treatment after CT	No. of observers	Actual treatment (follow-up)
Functional	Intra-articular distal radius fracture	Cast	2	Cast
Functional	Intra-articular distal radius fracture	Cast	3	Cast
Functional	Scaphoid fracture	Cast	2	Cast
Functional	Scaphoid fracture	Cast	1	Cast
Functional	Capitate fracture	Cast	3	Functional
Functional	Triquetral fracture	Cast	3	Cast
Functional	Triquetral fracture	Cast	3	Cast
Functional	Triquetral fracture	Percutaneous pin	1	Cast
Functional	None	Cast	1	Cast
Functional	None	Cast	1	Cast
Functional	None	Cast	1	Cast
Cast	Triquetral fracture in addition to a distal radius fracture	Cast, reduction	1	Cast
Cast	None	Cast, reduction	2	Cast, reduction
Cast	None	Cast, reduction	1	Cast, reduction
Cast	Intra-articularity distal radius fracture	Operative	1	Cast
Cast	Triquetral fracture in addition to an intra-articular distal radius fracture	Operative	1	Cast
Cast	None	Operative	1	Percutaneous pin
Cast	None	Operative	1	Open reduction and fixation
Cast	None	Operative	1	Cast
Cast	None	Operative	2	Cast
Cast	None	Operative	1	Cast^a^
Cast	None	Operative	1	Cast

Each line represents a patient with a treatment upgrade (= more invasive treatment after CT as compared to before CT) according to at least one observer. This table also displays the additional findings on CT as compared to CR (if applicable) and actual treatment given to the patient at follow-up

^a^Patient lost to follow-up

**Table 3:** Treatment downgrades according to one or more observers

No. of patients	Treatment before CT	Treatment after CT	No. of observers	Real treatment
11	Cast	Functional	3	Functional in 4 patients Cast 7-12 days in 7 patients
3	Cast	Functional	2	Functional in 2 patients Cast 6 days in 1 patient
4	Cast	Functional	1	Functional in 3 patients Cast 13 days in 1 patient
1	Cast	Functional	1	Cast 44 days^a^
1	External fixation with reduction	Reduction and cast	1	Reduction and cast 30 days
1	Percutaneous pin with reduction	Cast	1	Cast and lost to follow-up

Each line represents a cluster of patients with no additional diagnosis on CT and treatment downgrades (= less-invasive treatment after CT compared to before CT) according to at least one observer. This table also displays the actual treatment given to the patient at follow-up, including the number of days in a cast

^a^Patient with a previous history of pseudoarthrosis of the scaphoid, but no acute fracture at the time of the study. This patient finally underwent an elective screw fixation after several weeks

In wrists without additional findings, one or more observers still upgraded treatment in 11 wrists (3-6 per observer, Table 2) because they were initially not convinced on presence of fractures, or they were unsure about doing reduction or operation based on CR findings.

Of all patients without additional findings, one or more observers downgraded treatment in 21 patients (21% of the total population, 95% CI: 14–30%, 15–17 patients per observer, Table 3), mainly refraining from cast (18 patients, 14–16 patients per observer). Four of these 21 patients did not receive targeted oblique CR views before CT. These patients had a false-positive fracture suspicion at CR of the radius (two), the ulna (one), and the scaphoid (one). Although none of the patients with downgrades according to the observers had fractures at follow-up, nine of them were treated with cast for a maximum of 12 days (Table 3).

## Discussion

In this prospective study, we studied a fast volume wrist CT as an investigation in all patients with clinical suspicion of wrist fractures. Single-shot CT had a much higher accuracy than CR, and increased fracture detection in 26% of wrists. In addition, CT increased radiologists’ and clinicians’ certainty on fracture detection and intra-articularity of distal radius fractures. These findings are concordant with previous studies with different CT protocols in selected population on distal radius fractures [11], and are better than in previous publications on carpal fractures [12]. The additional fractures mainly included a higher number of (avulsion) fractures of the proximal carpal row, with a higher accuracy for carpal injury than previously published [12]. Recent studies have described this pattern of additional diagnoses also for cone beam CT [17] and even for sonography [18]. However, in contrast to most of these studies on selected patient groups, this was the first study that prospectively investigated CT as an imaging tool in *all* patients with clinical suspicion of wrist fractures, with a multiobserver study on treatment impact.

We found therapeutic changes in a substantial proportion (varying between 24 and 31% for different observers) and we saw a trend towards better interobserver agreement on treatment planning after CT diagnosis. This is concordant with other studies on variations in wrist fracture treatments in selected populations, especially with less experienced observers [19, 20], but this is discordant with a study that compared CT to traction CR and did not find a significant impact on treatment choices, again in a selected patient population [21].

Upgrades occurred in a heterogeneous group, mainly due to changes from functional- to cast treatment because of additional carpal or radial fractures. Only four of these upgrades were due to potentially clinically significant missed fractures on CR: Two intra-articular distal radius fractures and two scaphoid fractures. The other missed carpal fractures or intra-articular fractures might not lead to a poorer long-term outcome: They have always been missed before the era of CT and MRI. Proposals for surgical treatment had a very low incidence in this study, with a large variability among observers and actual treatment, comparable to similar studies [1, 22].

The most homogenous and largest patient group with treatment changes consisted of those without additional or relevant findings and with treatment downgrades: CT ruled out fractures with higher confidence than CR, especially of the carpal bones. Observers therefore downgraded treatment from cast to functional treatments in 14-16% of all patients. These results suggest that the fear of missing fractures should diminish with the use of this type of CT, and that unnecessary cast immobilization or additional imaging can be prevented. This is in line with previous studies that suggest the cost-effectiveness of immediate exclusion of carpal injury with MRI or CT, preventing unnecessary cast immobilization and excluding carpal injury with acceptable reliability [23, 24]. So when it comes to advanced imaging in all patients with wrist injury suspicion, it is not the additional information of CT compared to CR in wrist fractures, but it is ruling out carpal injury that will really add benefit to the patients.

The additional costs of CT compared to CR mainly comprise a longer CT review time for the radiologist and higher costs of data storing capacity for the radiology department. However, technologist- and patient time are similar, and in our centre even shorter for CT than for CR. Finally, although effective radiation dose of CT (0.01–0.02 mSv) is higher than CR (< 0.005 mSv), it is still very low, the equivalent of only one or two days of natural background radiation.

This study has limitations. First, this was a single-site study with a heterogeneous patient group. A large group of patients were excluded: Although some patients were excluded randomly (because of a shutdown of the scanner) and informed consent needed to be present before CR, failure to get informed consent might have led to systematic selection bias. Second, we chose to study a heterogeneous group of patients reflecting clinical practice, as prospective discrimination between radial and carpal injury can be challenging in daily practice. Third, the main outcome, impact on patient treatment, is very difficult to measure. We therefore considered the treatment plans from emergency physicians in clinical practice not definite enough to be part of the main outcome parameter of the study. The observer study guaranteed a more controlled set up, with experienced observers with equal access to patient information, but still demonstrated differences in surgeon preferences, in line with previous publications [20]. Fourth, we used a standard of reference both including clinical follow-up and questionnaires, but we had a low response rate to the questionnaires and no standard patient visit during follow-up. This might have led to incorporation bias with an overestimation of CT accuracy: CT was both an index test but also had a large effect on fracture presence assessment at follow-up. However, we do not think that this largely affected the primary study outcome, as it is those patients with persisting complaints who completed the questionnaires.

Finally, not all patients underwent an oblique view in addition to an anteroposterior and a lateral CR view of the wrist. Although this can potentially decrease the advantage of CT, this could only have been the case in two patients in this study: In one patient with a missed scaphoid fracture without clinical suspicion of carpal injury with a treatment upgrade, and in one patient with a missed trapezoid fracture without a treatment upgrade. The other patients with relevant study outcomes already underwent a third view (especially in the downgrade group), had subtle intra-articular fracture components, nondisplaced fractures, or carpal fractures that will not be seen on a an additional view if they are not visible on anteroposterior and lateral images.

This study investigated the effect of CT performance on therapeutic choices. This is a surrogate outcome, since ultimately, the effect on patient outcome should be investigated, in order to study CT as a serious and potentially better alternative to CR in primary imaging of wrist trauma. For this, a randomized control trial would be needed in a more specific patient group, measuring patient outcome with follow up including patient visits and questionnaires. In such a, preferably multi-site, study, an improvement of 11.5 on the PRWHE score would be relevant [25]. However, given the high heterogeneity in questionnaire completion and a large difference between surgical and patient preferences, a very large group of patients would be needed. Potentially the group of patients with suspected carpal injury who might benefit from immediately ruling out fractures is the most interesting and easily accessible group of patients to investigate.

## Conclusions

In summary, single-shot volume CT is highly accurate in fracture detection at a relatively low radiation dose. Compared to radiography, CT has a higher detection rate, rules out fractures with greater confidence and changes treatment of patients with suspicion of wrist fractures, possibly avoiding unnecessary cast treatment.
